# Computational and NMR Conformational Analysis of Galactofuranoside Cycles Presented in Bacterial and Fungal Polysaccharide Antigens

**DOI:** 10.3389/fmolb.2021.719396

**Published:** 2021-08-25

**Authors:** Alexey G. Gerbst, Vadim B. Krylov, Nikolay E. Nifantiev

**Affiliations:** Laboratory of Glycoconjugate Chemistry, N.D. Zelinsky Institute of Organic Chemistry RAS, Moscow, Russia

**Keywords:** substituted galactofuranosides, conformations, DFT, MP2, shielding constants

## Abstract

Unlike pyranoside cycles which are generally characterized by strictly defined conformational preferences, furanosides are flexible and may adopt a wide range of available conformations. During our previous studies, conformational changes of galactofuranoside cycles upon total sulfation were described computationally, using a simple Hartree–Fock (HF) method, and principal conformers of the 5-membered galactose ring were revealed. However, in the case of more complex disaccharide structures, it was found that this method and the widely applied DFT-B3LYP produced results that deviated from experimental evidence. In this study, other DFT functionals (PBE0 and double hybrid B2PLYP) along with RI-MP2 are employed to study the conformational behavior of the galactofuranoside ring. Reinvestigation of galactofuranosides with a lactic acid substituent at O-3 revealed that changes in the orientation of lactic acid residue at O-3 might induce conformational changes of the furanoside cycle. Such findings are important for further modeling of carbohydrate–protein interaction.

## Introduction

The understanding of the 3D structure of biologically important oligosaccharide sequences and of their conformational mobility is required for the assessment of their immunodeterminant fragments and prediction of the topology of oligosaccharide binding to cellular receptors and lectins (Zhang et al., 2017). In this context, galactofuranoside containing oligosaccharide determinants which are often presented in the antigenic polysaccharide chains of pathogenic bacteria and fungi represents a special interest because of its biological importance (Richards and Lowary, 2009; Krylov and Nifantiev, 2020). In particular, galactofuranoside units were discovered as structural components of polysaccharides of *Klebsiella* (Rollenske et al., 2018; Argunov et al., 2019; Whitfield et al., 2020), *Enterococcus* (Krylov et al., 2015; Laverde et al., 2020), *Mycobacteria* (Bhamidi et al., 2011; Lowary, 2016), *Aspergillus* (Costachel et al., 2005; Kudoh et al., 2015; Krylov et al., 2018), *Cryptococcus* (Previato et al., 2017), and other pathogens.

**FIGURE 1:**
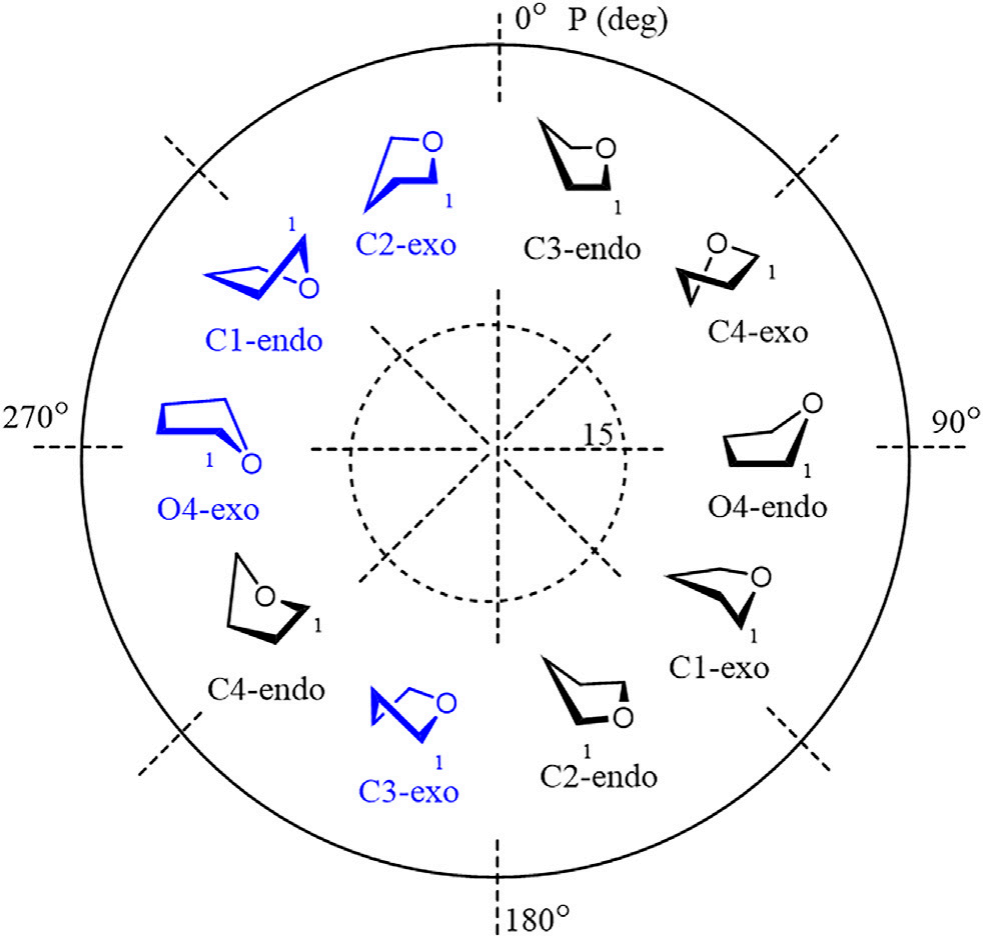
Main investigated conformers of the galactofuranoside ring (in blue).

Furanoside rings are generally considered to be more flexible than pyranosides. In our previous studies of the conformational changes in furanoside rings upon their complete sulfation, we revealed the main conformers of the non-sulfated galactofuranoside. These results were supported by evidence from NMR spectroscopy, such as intra-ring H–H coupling constants and NOE (Gerbst et al., 2019a). The main determined conformers for the non-sulfated rings were C3-*exo* or the ones similar to them according to Cremer–Pople parameters (Figure 1). The minor conformer in the case of the non-sulfated furanosides was O4-*exo* (Figure 1) which, however, became dominant upon the introduction of sulfates. The computational method employed in the referenced study was the HF/6-311++G** level of theory. Also, we employed the same method to study the driving force of the pyranoside-into-furanoside rearrangement (Gerbst et al., 2019b).

**FIGURE 2:**
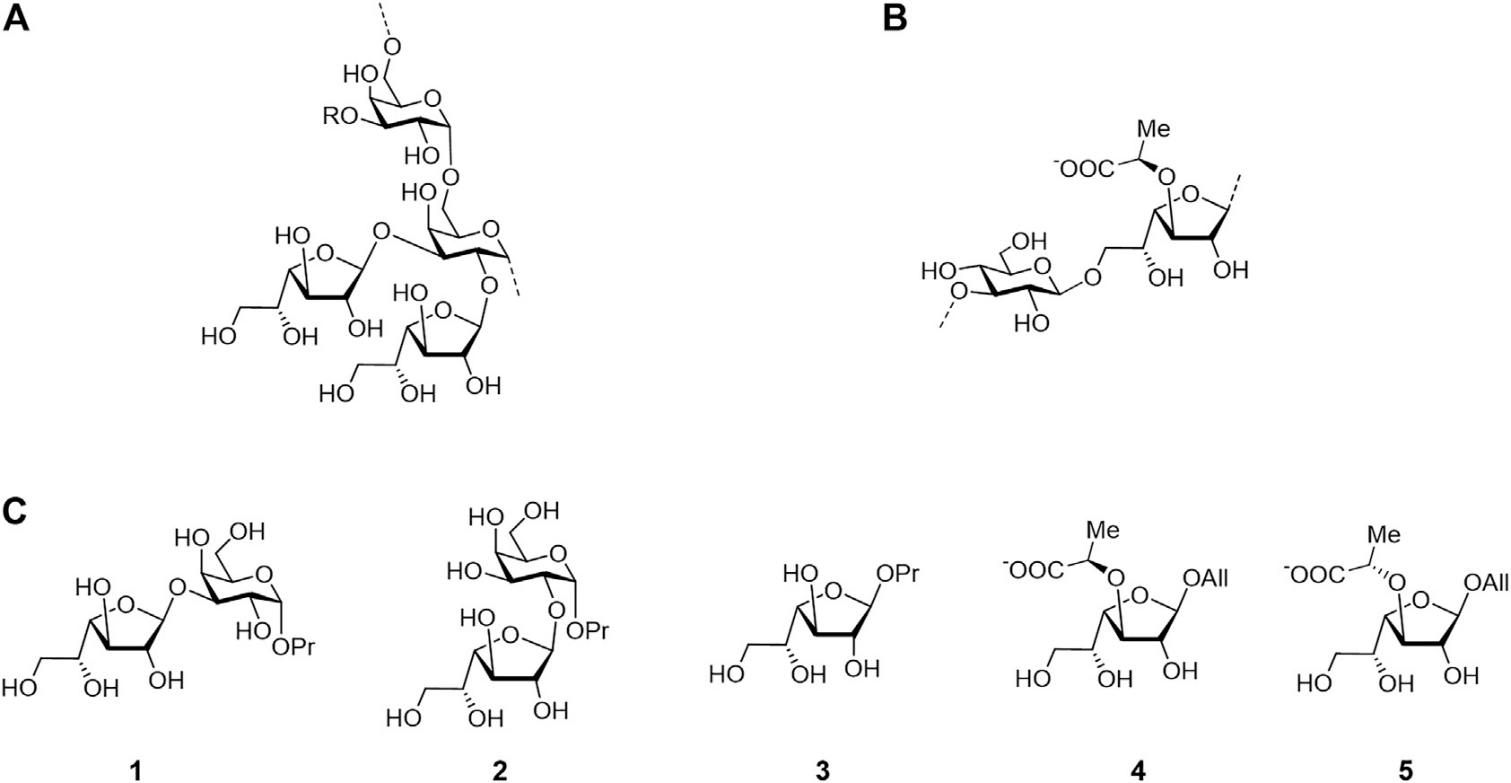
Structure of the *Cryptococcus neoformans* galactoxylomannan backbone **(A)**, *Enterococcus faecalis* diheteroglycan **(B)**, and related mono- and disaccharides studied in this work **(C)**.

However, in a further study (Dorokhova et al., 2021), we found that this approximation may not always produce correct results when computing the relative energies of the furanoside ring conformation. In the mentioned work, the conformations of furanoside rings in di- and trisaccharides were studied, and the results on their dependence upon the conformation of the glycosidic linkage were summarized. Herein, we attempted a detailed analysis of the application of various approximations to the energy analysis of furanoside conformers. The objects of the study are shown in Figure 2. Disaccharides 1 and 2 (that relate to the fragments of *Cryptococcus neoformans* galactoxylomannan) are model structures lacking an amino group in the propyl aglycon; synthesis of the full structures is described in the study by (Dorokhova et al., 2021), monosaccharide 3 is from the study by (Gerbst et al., 2019a), and synthesis of 3-O-(R)-lactic acid derivative 4 (that relates to the fragment of *Enterococcus faecalis* diheteroglycan) and its (S)-isomer 5 is described in the study by (Krylov et al., 2015).

## Materials and Methods

ORCA (Neese, 2012) (version 4.2) software was used throughout the study. DFT calculations employed either the 6-31G (d,p) (Hehre et al., 1972), 6-311++G**, or def2-TZVP (Weigend and Ahlrichs, 2005) basis sets with the B3LYP or PBE0 functional (see details in the text). Grimme dispersion correction was employed where noted in the text. All RI-B2PLYP and RI-MP2 calculations were carried out using the def2-TZVP basis set. NMR shielding constants were computed using DFT/B3LYP/def2-TZVPP (Weigend and Ahlrichs, 2005) approximation with the GIAO option and the automatic auxiliary JK basis set (Stoychev et al., 2017). The conductor-like polarizable continuum model (CPCM) of solvation (Barone and Cossi, 1998) with parameters for water was used to account for bulk solvent effects. No counter-ions were considered in the calculations.

## Results

The problem that inspired this investigation first occurred in the study by (Dorokhova et al., 2021) when we started the conformational analysis of model disaccharides 1 and 2. When the use of the previously approbated method, HF/6-311++G**, was attempted, it was found that the C3-*exo* conformer was predicted to be more preferable than O4-*exo* by the order of 2 kcal/mole. This contradicted the experimentally observed intra-ring ^1^H–^1^H couplings. Additionally, when the computational methods varied, sometimes during torsional scans on the glycosidic linkages in these disaccharides, we encountered a situation that could be determined to be “a conformational hell”: the desired O4-*exo* conformer often irreversibly transformed into C3-*exo* and never *vice versa* (Table 1).

**TABLE 1:** Conversion of different conformers during geometry optimizations of disaccharide **1**.

Method	Starting conformer	Resulting conformer
HF/6-311++G**	O4-*exo*	O4-*exo*
B3LYP/6-311++G**	O4-*exo*	C3-*exo*
B3LYP/6-31G (d,p)	O4-*exo*	C3-*exo*
HF/6-31G (d,p)	O4-*exo*	C1-*endo*
RI-MP2/def2-TZVP	O4-*exo*	O4-*exo*
B2PLYP/def2-TZVP	O4-*exo*	O4-*exo*
HF/6-311++G**	C3-*exo*	C3-*exo*
B3LYP/6-311++G**	C3-*exo*	C3-*exo*
RI-MP2/def2-TZVP	C3-*exo*	C3-*exo*
B2PLYP/def2-TZVP	C3-*exo*	C3-*exo*

**FIGURE 3:**
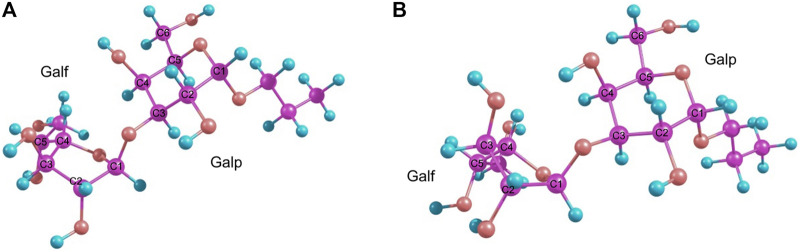
Main conformers of disaccharide **1** as obtained from RI-MP2 calculations: O4-*exo* **(A)** and C2-*exo* **(B)**.

Finally, it was found that the RI-MP2/def2-TZVP approach provided satisfactory results. This method was chosen as being of the higher level of theory since it takes into account the electronic correlation. The main conformers found for these disaccharides finally turned out to be O4-*exo* and C2-*exo* (Figure 3). This suggested re-investigation of the furanoside ring conformations in the simple propyl-galactofuranoside 3. A variety of methods (including hybrid B3LYP and PBE0 and double hybrid B2PLYP DFT functionals along with the RI-MP2 approach) was applied to conduct geometry optimization of its C3-*exo* and O4-*exo* conformers. The results are presented in Table 2.

**TABLE 2:** Electronic energies (a.u.) obtained for conformers of monosaccharide **3** using different methods.

Method	Electronic energies of conformers (a.u.)		ΔE (kcal/mole)
	C3-*exo*	O4-*exo*	
B3LYP/6-31G (d,p)	−804.6851292	-^a^	n.a
PBE0/6-31G (d,p)	−804.2509974	-^a^	n.a
PBE0/def2-TZVP	−804.5633591	-^a^	n.a
B3LYP/6-31G (d,p)/D3	−804.7357417	−804.7351638	0.4
B2PLYP/def2-TZVP	−804.8280619	−804.8266101	0.9
RI-MP2/def2-TZVP	−803.6819645	−803.6816517	0.2

a Conversion into the C3-exo conformer during optimization occurred.

Additionally, we compared the lengths of endocyclic C–C and C–O bonds for the C3-*exo* conformer of this compound computed using different methods with those obtained using the X-ray method for a galactofuranoside containing disaccharide (PDB entry 4XAD). The results are presented in Table 3. It can be seen that all the carbon–carbon bonds are by several hundredths of an angstrom (but not more than 0.05 Å). Meanwhile, all the C–O bonds are slightly underestimated. We attribute this discrepancy to the fact that the experimental values were obtained in the crystal, while the calculations were carried out in bulk water using the CPCM model.

**TABLE 3:** Comparison of experimental (PDB entry 4XAD) and computed bond lengths for the C3-*exo* conformation of monosaccharide **3** (Å).

Method	C1-C2	C2-C3	C3-C4	C4-O4	O4-C1
Experimental	1.50642	1.49672	1.51769	1.44631	1.44390
B3LYP/6-31G (d,p)	1.53746	1.52996	1.53765	1.43834	1.42992
PBE0/6-31G (d,p)	1.53055	1.52182	1.52912	1.42569	1.41786
PBE0/def2-TZVP	1.52493	1.51683	1.52533	1.42365	1.41698
B3LYP/6-31G (d,p)/D3	1.53790	1.52939	1.53460	1.43574	1.42562
B2PLYP/def2-TZVP	1.52828	1.52182	1.52907	1.43494	1.42670
RI-MP2/def2-TZVP	1.52645	1.51571	1.52326	1.43259	1.42558

The electronic energies obtained for both conformers are the same for B3LYP and both PBE0 approaches. This occurs because both these methods indeed transform the O4-*exo* conformer into the C3-*exo*, like it was the case for structures 1 and 2. When the dispersion correction (D3) is applied, the starting conformation does not change during the geometry optimization, and the C3-*exo* conformer is predicted to be 0.4 kcal/mole more preferable.

**FIGURE 4:**
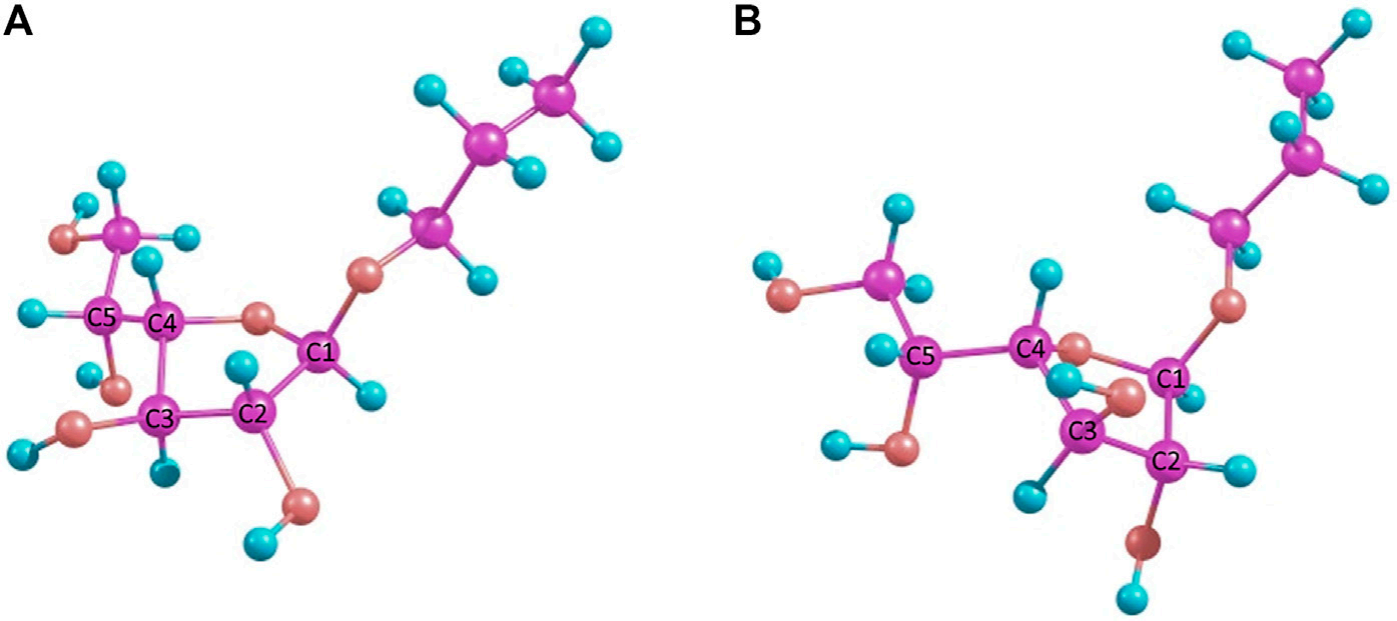
MP2-optimized conformers of the simple β-propyl-galactofuranoside **3**: C3-*exo* **(A)** and C4-*endo* **(B)**.

For the double-hybrid B2PLYP DFT and pure MP2, however, the final energy values differ, which reflects the fact that the initial ring conformation is retained. These differences in the table values correspond to 0.9 kcal/mole for B2PLYP and 0.2 kcal/mole for MP2, while the C3-*exo* conformer is still more preferable. The resulting conformers obtained at the MP2 level are shown in Figure 4.

Next, we performed a re-investigation of monosaccharides 4 and 5, which are allyl galactofuranosides substituted at O-3 with a lactic acid residue having an R- or S-configuration. Such units are present in a capsule polysaccharide (diheteroglycan) of *Enterococcus faecalis.* The same scope of methods was applied for these molecules (Table 4 and Table 5). The resulting energies for each isomer are presented in Table 4 and Table 5. One of the most important features of these two structures is the orientation of the lactic acid substituent whose tertiary proton (H-Lact) can be spatially closer either to the H-2, H-4, or H-3 proton of the furanoside ring. The difference in its orientation helps to establish absolute configuration of the lactic acid (Krylov et al., 2015).

**TABLE 4:** Relative energies (kcal/mole, against the lowest energy conformer marked in bold) for principal conformers of the D-lactic acid derivative **4**) of the furanoside ring calculated at different levels of theory.

Conformer	Levels of theory					
	B3LYP/6-31G (d,p)	PBE0/6-31G (d,p)	PBE0/def2-TZVP	B3LYP/6-31G (d,p)/D3	B2PLYP/def2-TZVP	RI-MP2/def2-TZVP
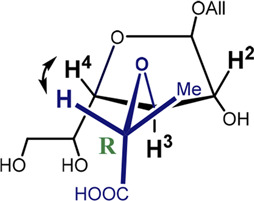 **H-4**—**H-Lact** proximity						
C3-*exo*	4.5	4.6	2.2	5.2	2.8	3.5
**C2-*exo***	**0.0**	**0.0**	**0.0**	**0.0**	**0.0**	**0.0**
O4-*exo*	3.1	2.7	1.8	2.9	2.0	1.8
C1-*endo*	3.2	2.8	1.8	2.6	2.2	1.9
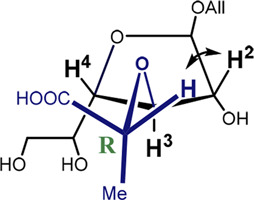 **H-2**—**H-Lact** proximity						
C3-*exo*	3.8	4.0	1.3	5.2	2.2	3.0
C2-*exo*	3.1	3.3	1.9	2.7	2.2	3.1
O4-*exo*	4.6	4.5	2.0	5.1	2.5	3.0
C1-*endo*	4.6	4.4	2.1	5.1	2.5	3.1
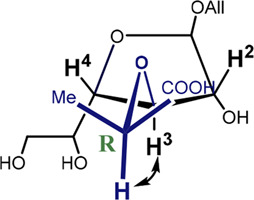 **H-3**—**H-Lact** proximity						
C3-*exo*	−4.8	−5.0	1.1	−4.7	0.9	0.3
O4-*exo*	-^a^	-^a^	-^a^	-^a^	1.5	1.4

a Conversion into the C3-exo conformer during optimization.

**TABLE 5:** Relative energies (kcal/mole, against the lowest energy conformer marked in bold) for principal conformers of the L-lactic acid derivative **5**) of the furanoside ring calculated at different levels of theory.

Conformer	Levels of theory					
	B3LYP/6-31G (d,p)	PBE0/6-31G (d,p)	PBE0/def2-TZVP	B3LYP/6-31G (d,p)/D3	B2PLYP/def2-TZVP	RI-MP2/def2-TZVP
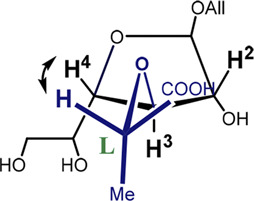 **H-4**—**H-Lact** proximity						
C3-*exo*	7.4	9.3	4.1	10.1	4.2	5.3
C2-*exo*	7.0	6.9	4.6	6.3	4.7	5.1
O4-*exo*	8.8	9.4	6.3	9.2	6.1	7.0
O4-*exo*	8.8	9.4	6.3	9.2	6.1	7.0
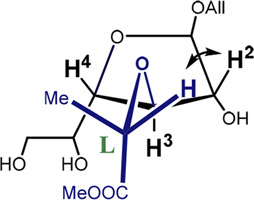 **H-2**—**H-Lact** proximity						
C3-*exo*	4.6	4.8	2.6	5.2	2.3	2.5
C2-*exo*	3.8	3.4	1.6	3.7	2.2	1.6
**O4-*exo***	**0.0**	**0.0**	**0.0**	**0.0**	**0.0**	**0.0**
C1-*endo*	3.0	3.2	1.2	3.5	0.9	0.4
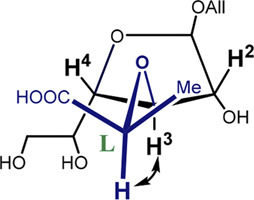 **H-3**—**H-Lact** proximity						
O4-*exo*	5.8	5.0	3.9	7.2	2.3	2.0
C2-*exo* ^a^	6.4	5.3	2.9	−0.6	3.1	2.5
C3-*exo*	9.5	10.2	10.0	7.3	6.7	5.9

a The lactic acid residue slightly rotated toward H-2.

**FIGURE 5:**
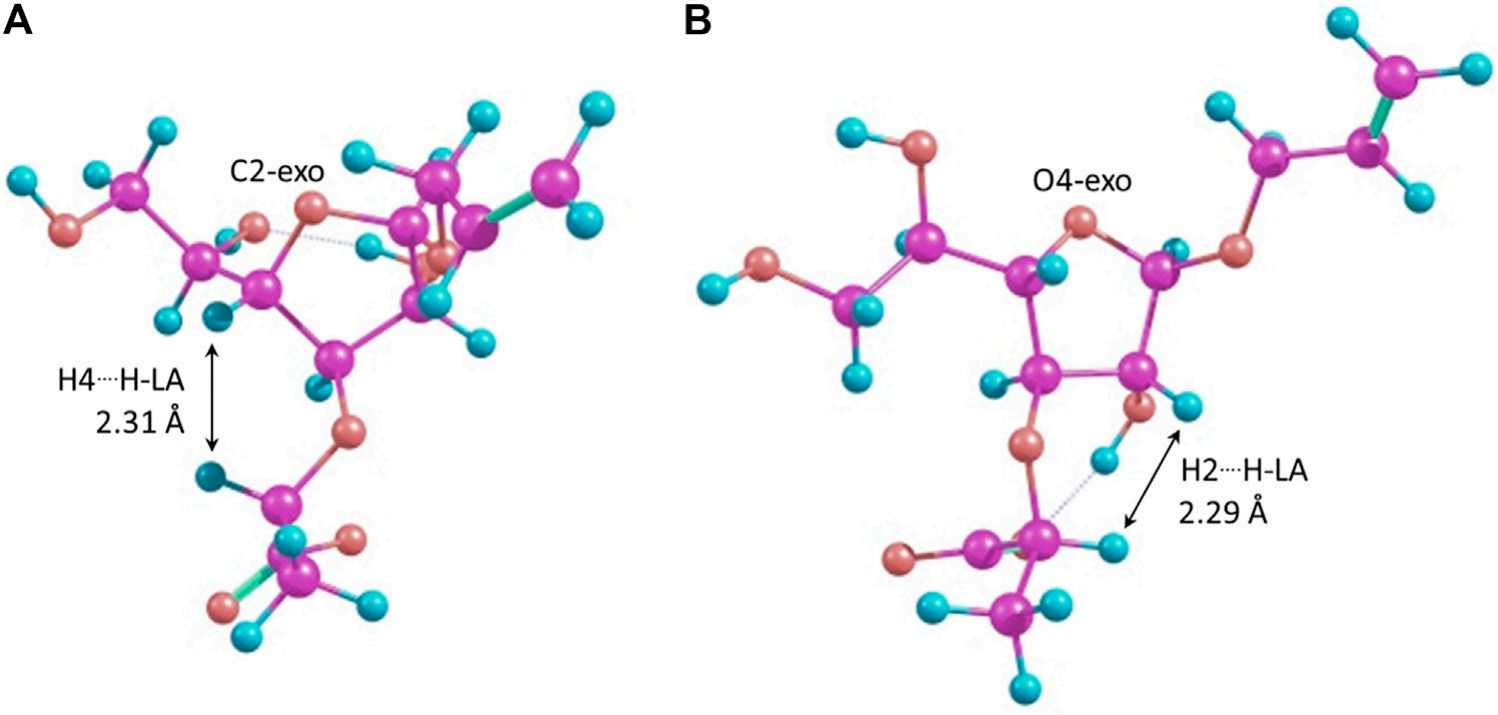
Major conformers of monosaccharides **4** **(A)** and **5** **(B)**.

It can be seen that the C2-*exo* conformer with the spatial proximity of the lactic acid proton to H-4 has the lowest energy for structure 4 (R-lactic acid derivative), while for structure 5, it is O4-*exo* with the lactic acid proton oriented toward H-2. Generally, its orientation toward H-2 is more pronounced in the latter molecule, while in structure 4, both orientations seem possible. Graphical representations of the lowest energy conformers with the inter-proton distances are given in Figure 5.

As for the conformers with the spatial proximity of H-Lact to the H-3 ring, proton conformational changes were detected that depended upon the computational method and the structure involved. In the case of molecule 4, all DFT methods (except double-hybrid B2PLYP) transformed the starting conformations, C1-*endo*, C2-*exo*, and O4-*exo*, to C3-*exo*. The other two methods rendered the O4-*exo* conformation as the result, except for the starting C3-*exo*, which was retained. In both cases, H-Lact and H-3 remained close to each other with the distances of ca. 2.20–2.24 Å. For S-isomer 5, C1-*endo* changed to O4-*exo*, keeping H-Lact and H-3-close, while O4-*exo* itself did not change. C2-*exo* did not change either, but the lactic acid residue slightly rotated toward H-2, distances from H-Lact to H-3 and H-2 being 2.36 Å and 3.26 Å, respectively. The C3-*exo* conformation did not change but had the largest energy. Meanwhile, for the listed conformers, the energies were sometimes below the lowest ones mentioned in Table 4 and Table 5.

After that, ^1^H NMR shielding constants were calculated for the obtained conformers except for the C3-*exo*/H-3 conformer of molecule 5, since it had significantly higher energy. The DFT B3LYP approach with a triple zeta basis with two polarization functions (def2-TZVPP) was applied because smaller basis sets can produce unreliable results for this task. As input structures for these calculations, RI-MP2–optimized conformations were used. These were chosen because the RI-MP2 approach is supposed to provide more accurate results. It was found that shielding constants for the anomeric proton, as a rule, did not change significantly. Knowing its experimentally measured chemical shift of 5.06 ppm (Krylov et al., 2015), a value of 31.65 was chosen as a shielding constant corresponding to a chemical shift of 0 ppm. The resulting chemical shifts along with the experimental ones for both compounds are given in Table 6 and Table 7, and the discussion on them can be found in the next section.

**TABLE 6:** ^1^H chemical shifts (ppm) obtained from the computed shielding constants for conformer structure **4** and the experimental ones marked in bold (Krylov et al., 2015).

Conformer/ring proton spatially close to the lactic acid proton	H-1	H-2	H-3	H-4	H-5	H-6	H-6′
C3-*exo*/H-2	5.02	4.16	4.33	3.94	4.11	3.96	3.99
C3-*exo*/H-4	5.38	4.50	3.80	4.16	4.19	4.69	3.87
C2-*exo*/H-2	5.06	3.97	4.14	4.48	4.12	3.94	3.95
C2-*exo*/H-4	5.05	3.92	4.16	4.71	4.00	3.95	3.94
C1-*endo*/H-2	5.16	4.19	3.66	3.80	3.93	4.05	3.63
C1-*endo*/H-4	5.15	4.32	3.82	3.96	3.66	4.08	3.61
O4-*exo*/H-2	5.15	4.20	3.67	3.81	3.93	4.02	3.59
O4-*exo*/H-4	5.17	4.33	3.91	3.99	3.62	4.05	3.56
C3-*exo*/H-3	5.09	4.34	3.44	3.88	3.96	3.96	3.76
O4-*exo*/H-3	5.00	5.14	3.36	3.76	3.85	3.92	3.69
**Experimental**	**5.06**	**4.25**	**3.9**	**4.07**	**3.86**	**3.72**	**3.67**

**TABLE 7:** ^1^H chemical shifts (ppm) obtained from the computed shielding constants for conformer structure **5** and the experimental ones marked in bold (Krylov et al., 2015).

Conformer/ring proton spatially close to the lactic acid proton	H-1	H-2	H-3	H-4	H-5	H-6	H-6′
C3-*exo*/H-2	5.00	4.36	3.81	3.79	3.91	5.57	3.49
C3-e*xo*/H-4	4.93	4.13	4.13	3.87	3.99	3.95	4.01
C2-*exo*/H-2	5.06	4.09	4.18	4.49	4.17	3.90	3.93
C2-*exo*/H-4	5.06	3.93	4.28	4.63	4.20	3.95	3.99
C1-*endo*/H-2	5.04	4.37	3.79	3.70	3.87	5.04	3.52
C1-*endo*/H-4	5.16	4.27	3.73	3.89	3.81	3.82	3.75
O4-*exo*/H-2	5.09	4.28	3.62	3.86	3.88	4.16	3.48
O4-*exo*/H-4	5.17	4.24	3.74	3.92	3.83	4.16	3.76
O4-*exo*/H-3	5.07	3.92	3.88	4.94	4.76	4.02	4.02
O4-*exo* *^a^*/H-3	5.12	4.30	3.79	3.88	4.02	5.83	3.49
**Experimental**	**5.06**	**4.25**	**3.91**	**4.08**	**3.85**	**3.73**	**3.66**

a The lactic acid residue slightly rotated toward H-2.

## Discussion

While conformations of pyranoside rings have been extensively studied and their conformational preferences are well known in general cases, furanosides represent a more complex subject due to their ring flexibility. A number of investigations are present that employ different quantum mechanical methods with various basis sets, but they are all focused primarily on the conformations of pentafuranosides [for a review, see (Taha et al., 2013)]. More often, conformations of furanoside rings were studied using methods of molecular mechanics. In the work by (Richards et al., 2013), the conformation of methyl galactofuranosides was studied, but the study was limited to the B3LYP functional with 6-31G* or 6-31+G**, and the investigated structures did not contain ring substituents. A recent work (Gaweda et al., 2020) was dedicated to the computational study of the exo- and endo-anomeric effect in furanoside rings. However, these authors employed a rather small basis set [6-31G(d)] but an advanced functional, M062X. This choice was justified because they use only simple model molecules for their study. Our results reported above demonstrate that when dealing with more complicated structures, much more strongly resembling those occurring in real biologically important molecules, conventionally used HF and DFT methods may not always work. For example, even in a simple propyl galactofuranoside 3, DFT methods without dispersion correction failed to reproduce the O4-*exo* conformation as a stationary point, converting it to C3-*exo*. Expectedly, usage of methods with more precise accounting for electronic correlation (the double-hybrid B2PLYP functional and RI-MP2) gives results similar to those obtained with D3 correction, although the energy values differ slightly (Table 2). Meanwhile, as was previously shown in the study by (Krylov et al., 2015), the presence of the O4-*exo* conformer is necessary to explain the observed intra-ring ^1^H–^1^H couplings. A similar effect was observed for structures 1 and 2, where more-or-less sensible conformational analysis was not possible without the use of MP2-based approaches. Of course, B3LYP/6-31G (d,p)/D3 outperforms MP2 methods in terms of time on the same computing resources. Probably, further investigations are needed to establish whether the use of the former approximation is always sufficient to model furanoside ring conformations.

**FIGURE 6:**
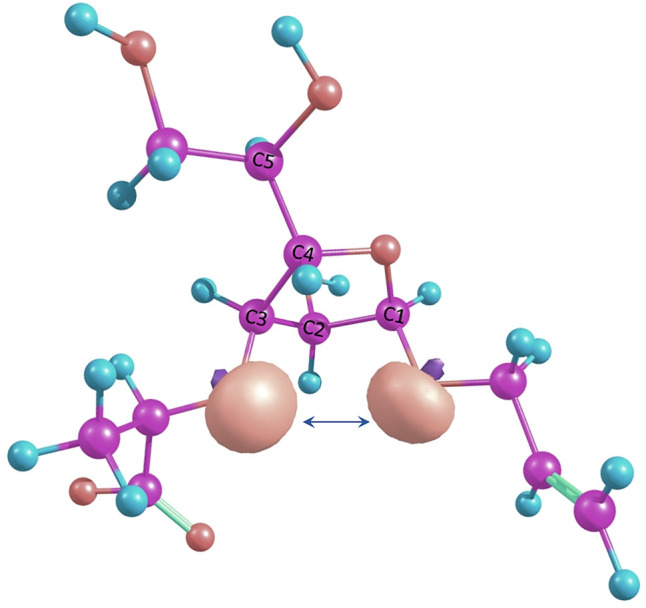
Suggested 1-3-*syn*-diaxial repulsion between O-3 and O-1 in the O4-*exo* conformation illustrated by NBO lone pairs.

Changes in furanoside ring puckering occurring during modeling of conformers with the spatial proximity of H-Lact and H-3 ring protons in structures 4 and 5 can be explained by the so-called 1-3-*syn-*diaxial repulsion. In these conformers, when O-3 and O-1 substituents are initially pseudo-axially oriented, lone pairs on the oxygen atoms become oriented in a way causing them to move away from each other. This is illustrated in Figure 6, showing these lone-pair orbitals as calculated using NBO6 software (Glendening et al., 2013). This results either in a ring conformation change or in rotation of the O-3 substituent. O-1 is less prone to rotation due to the anomeric effect. This is confirmed by the fact that the C3-*exo* conformation is always retained.

This effect is also encountered in pyranosides. For example, in the work by (Komarova et al., 2017), we observed that this effect can cause conformational interchanges in idopyranosides. Presumably, in furanosides, it may influence to a larger extent since these molecules are not as conformationally rigid as pyranosides.

For structures 4 and 5, the calculations demonstrate that the furanoside ring conformation does not affect the preferable orientation of the 3-O-lactic acid substituent. For the R-isomer contacts between H-Lact and H2 and H4, protons are observed for each of the considered conformers. For the S-isomer, a strong preference for H-Lact/H2 contact is demonstrated. Energy values allow the suggestion that R-isomer 4 might exist preferably with H-Lact in proximity to H-3 with the possibility to rotate either toward H-2 or toward H-4, which is accompanied by the ring conformation change. For S-isomer 5, conformations in which H-Lact is in proximity to H-4 are not likely to give a significant contribution. This explains the previously observed NOE (Krylov et al., 2015) and confirms that the absolute configuration of the lactic acid substituent was determined correctly.

NMR techniques are a powerful tool for conformation analysis of carbohydrates (Lipkind et al., 1992; Grachev et al., 2009). In the described case, we performed analysis of the computed chemical shifts. The computed values presented in Table 6 and Table 7 do not allow us to determine definite trends in them upon the conformational changes. The anomeric H-1 chemical shift is probably the most conserved one. The wide range of computed chemical shifts for H-6 protons is most likely due to their dependence upon the side chain conformation. Some values for H-2, H-3, and H-4 computed chemical shifts (e.g., C3-*exo*, C1-*endo*, and O4-*exo* in Table 7; C3-*exo* and C2-*exo* in Table 7) have shown that these shifts rather depend upon the lactic acid residue orientation. This situation cannot be called unexpected, as the same can be found in pyranosides, too, but the absence of any pronounced changes upon the change in ring puckering makes these structures different from six-membered sugar rings, in which even a slight distortion can cause considerable shift changes (Gerbst et al., 2018).

## Conclusion

Several DFT and MP2-based methods were compared in modeling of furanoside ring conformations, including ones with a lactic acid substituent. It was found that at least dispersion correction should be applied to achieve sensible results. MP2 methods also perform well, although they are certainly more time-demanding. It is demonstrated that changes in the orientation of the lactic acid residue at O-3 induce conformational changes of the furanoside cycle. Meanwhile, the unsubstituted propyl galactofuranoside exhibits just two primary conformers. These ring conformational changes occur upon the substituent rotation, most likely through the 1-3-*syn*-diaxial repulsion. This should be kept in mind since in pyranosides, this repulsion can also occur but is not considered to affect the conformation in general cases. This might also mean that the range of adoptable conformations for the furanoside rings may depend upon the substation pattern. It is shown that while in pyranosides, ^1^H NMR chemical shifts considerably depend on the conformational distortion, this is not the case for furanosides, where these shifts rather depend on a ring substituent orientation.

Considering the ongoing progress of computational methods, particularly in the structural analysis of carbohydrates (Gerbst et al., 2021), these results may present interest for further investigation of the biologically important furanoside-containing compounds.

## Data Availability

The original contributions presented in the study are included in the article/Supplementary Material; further inquiries can be directed to the corresponding authors.
